# Supplementary material for: The interactome of intact mitochondria by cross-linking mass spectrometry provides evidence for coexisting respiratory supercomplexes
Source: Mol Cell Proteomics. 2017 Dec 8;17(2):216–32. doi: 10.1074/mcp.RA117.000470 (PMC5795388; doi:10.1074/mcp.RA117.000470)
Supplement: Supplemental Data [file supp_RA117.000470_133922_0_supp_23978_fzffwf.zip › spectra_annotation/mito_DR_spectra_annotation/59-2-1-1-8-1.pdf]

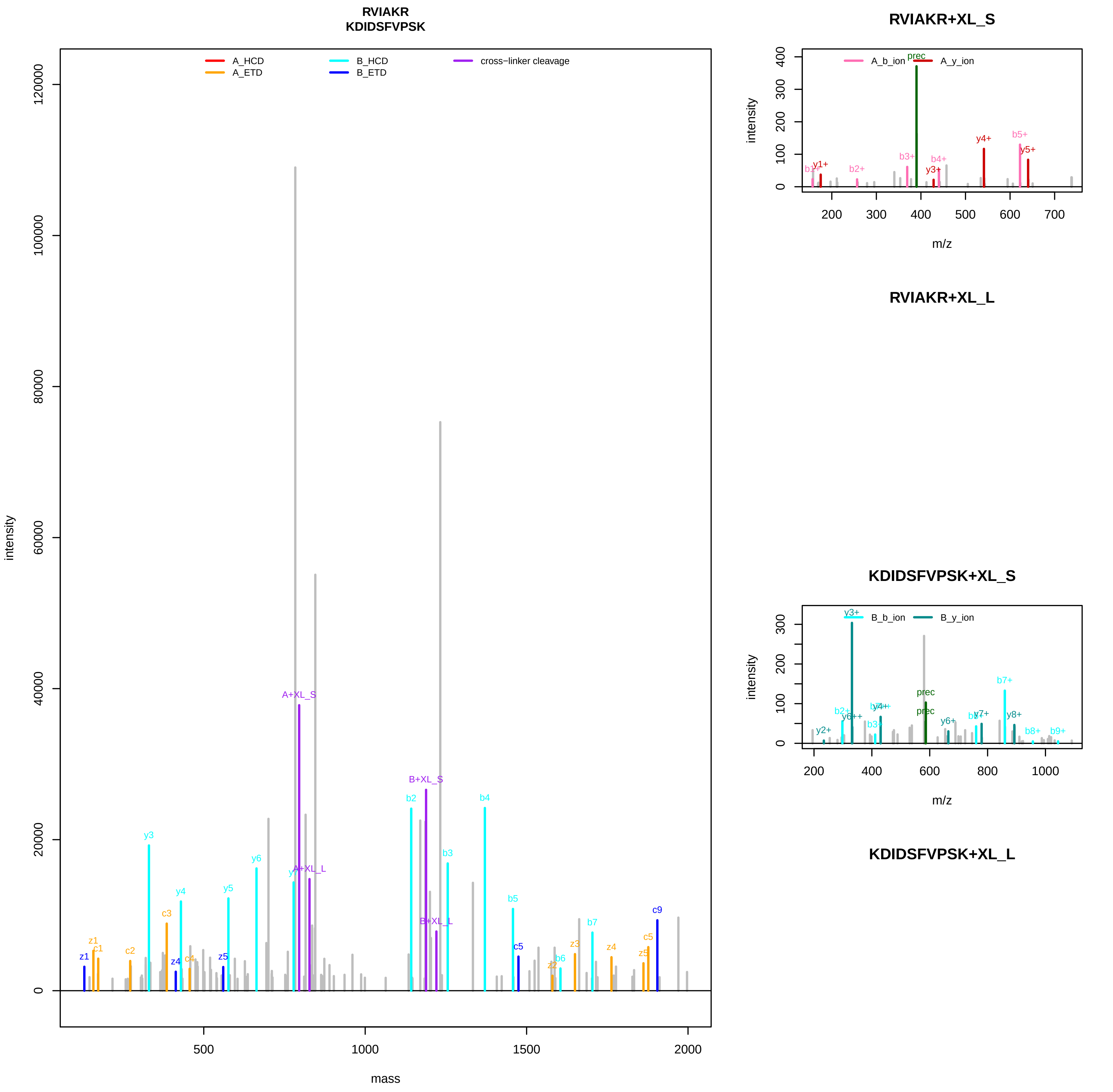

RVIAKR
KDIDSFVPSK
A_HCD
A_ETD
B_HCD
B_ETD
cross−linker cleavage
intensity
mass
0
20000
40000
60000
80000
100000
120000
500
1000
1500
2000
z1
z1
c1
c2
y3
c3
y4
z4
c4
z5
y5
y6
A+XL_S
A+XL_L
b2
B+XL_S
B+XL_L
b3
b4
b5
c5
z2
b6
z3
b7
z4
z5
c5
c9
RVIAKR+XL_S
A_b_ion
A_y_ion
prec
m/z
RVIAKR+XL_L
KDIDSFVPSK+XL_S
B_b_ion
B_y_ion
KDIDSFVPSK+XL_L
